# Diagnostic Utility of Skin Prick Test Ratios and Specific IgE in Predicting Egg White Allergy: Reducing the Need for Oral Food Challenges in Children

**DOI:** 10.3390/jcm15041382

**Published:** 2026-02-10

**Authors:** Filiz Demir Şahin, Ozan Kapçay, Mehmet Kılıç, Hilal Şahin Sindi

**Affiliations:** Department of Pediatric Allergy and Immunology, Fırat University, 23119 Elazığ, Turkey; okapcay@firat.edu.tr (O.K.); mkilic@firat.edu.tr (M.K.); hssindi@firat.edu.tr (H.Ş.S.)

**Keywords:** egg white allergy, oral food challenge, skin prick test, prick/histamine ratio, specific IgE

## Abstract

**Background**: Skin prick testing (SPT) and serum egg white–specific IgE (sIgE) support oral food challenge (OFC) decisions in suspected egg allergy, but the incremental value of histamine-normalized SPT indices remains uncertain. **Methods**: In this single-center retrospective study, 105 egg-sensitized children underwent clinically indicated OFC. Commercial egg white SPT, prick-to-prick testing with fresh egg white, histamine controls, and serum egg white–specific IgE were assessed. Discriminatory performance was evaluated by ROC analysis, and independent predictors of OFC positivity were identified using multivariable logistic regression. **Results**: OFC was positive in 23 of 105 children (21.9%). Egg white SPT wheal diameter, the prick-to-histamine ratio, and serum egg white–specific IgE (sIgE) levels were significantly higher in OFC-positive patients (all *p* < 0.001). ROC analysis demonstrated moderate-to-good discrimination for both sIgE (AUC = 0.767) and the prick-to-histamine ratio (AUC = 0.786), without clear superiority of normalized indices. In multivariable logistic regression analysis, only absolute egg white SPT wheal diameter (adjusted OR 1.41 per 1 mm increase; 95% CI 1.13–1.77) and serum egg white–specific IgE level (adjusted OR 31.86 per 100 kUA/L increase; 95% CI 1.60–636.15) remained independent predictors of OFC positivity. **Conclusions**: Absolute egg white SPT wheal diameter and serum sIgE independently predict OFC outcomes in egg-sensitized children. Histamine-normalized indices did not provide added value over wheal size alone. These findings support a probabilistic, context-based use of test results to inform selective OFC planning rather than replace OFC.

## 1. Introduction

Food allergies are a common pediatric health problem, predominantly affecting early childhood, with substantial adverse effects on quality of life and posing significant challenges in diagnostic evaluation and clinical management [1,2]. Egg allergy, together with cow’s milk allergy, is among the most frequently implicated food allergens in children, with a reported prevalence of approximately 1–3% during the first years of life [3,4,5]. Although tolerance develops over time in a substantial proportion of patients, diagnostic uncertainty may result in unnecessary dietary restrictions and increased parental anxiety.

The oral food challenge (OFC) remains the gold standard for confirming clinical reactivity; however, concerns regarding anaphylaxis risk and its time- and resource-intensive nature limit its routine use in clinical practice [1,6]. Consequently, optimizing the diagnostic performance of non-invasive biomarkers, including skin prick testing (SPT) and serum egg white–specific immunoglobulin E (sIgE), is essential—not to replace oral food challenges (OFCs), but to support more rational and selective challenge planning for predicting clinical reactivity [6,7,8,9,10,11]. Nevertheless, threshold values for SPT and sIgE reported in the literature vary considerably across populations, thereby limiting the clinical utility of universal cut-off points [6,7,8,9,10,11]. This heterogeneity is thought to be partly attributable to technical variability in SPT application as well as interindividual biological differences.

To address these limitations, normalized SPT indices such as the prick-to-histamine ratio have been proposed. However, whether these parameters provide incremental diagnostic value over absolute wheal diameters in predicting clinical reactivity remains insufficiently clarified [7,8,9,10,11]. Therefore, the aim of the present study was to directly compare the discriminatory performance of egg white SPT wheal diameter, prick-to-histamine ratio, and serum sIgE levels in predicting OFC positivity among egg-sensitized children in a pediatric population. By providing accessible and population-specific data applicable even in clinical settings with limited access to advanced molecular diagnostics, this study seeks to support a more balanced diagnostic approach—one that does not aim to replace OFC, but rather to reduce unnecessary procedures through improved risk stratification.

## 2. Materials and Methods

### 2.1. Study Design and Population

This single-center, retrospective observational study was conducted by reviewing the medical records of pediatric patients who presented to the Pediatric Allergy and Immunology Clinic at Fırat University between January 2019 and July 2024 for evaluation of suspected egg white allergy. The study cohort comprised 105 children with comprehensive clinical documentation who had undergone skin prick testing (SPT) for egg white according to standardized protocols and oral food challenge (OFC) with egg based upon clinical indication.

Exclusion criteria encompassed patients with well-documented histories of egg-induced anaphylaxis (for whom challenge testing was contraindicated), individuals with confirmed immunodeficiency disorders, and those presenting with concurrent acute infectious processes at the time of OFC administration.

### 2.2. Skin Prick Testing Procedures

Skin prick testing (SPT) was performed on the volar surface of the forearm using standardized commercial egg white allergen extracts (Asacpharma, ApiPrick^®^, Barcelona, Spain) applied with single-use disposable applicators (Medblue Allergy, Asistan Medikal, Gaziantep, Turkey). Histamine dihydrochloride (10 mg/mL) and 0.9% sodium chloride solution (normal saline) were used as positive and negative controls, respectively. Wheal diameters were measured in millimeters at 15 min. The largest diameter (D1) and the orthogonal diameter (D2) were recorded, and the mean wheal diameter was calculated as (D1 + D2)/2. A positive SPT response was defined as a wheal diameter ≥ 3 mm greater than that of the negative control. Prior to oral food challenge, skin prick testing and serum egg white–specific IgE measurements were performed within a clinically acceptable and short time frame. In most patients, SPT and sIgE assessments were performed within 2–3 weeks prior to OFC, and in all cases within 1 month. Antihistamine medications were discontinued for appropriate time intervals prior to testing, in accordance with current clinical guidelines.

Prick-to-prick testing was incorporated into our diagnostic workflow as a complementary modality to mitigate potential false-negative results associated with commercial extracts. Although applied routinely as part of a standardized institutional protocol, prick-to-prick testing was not used as a screening tool or in isolation; all findings were interpreted in conjunction with standard skin prick testing, serum egg white–specific IgE measurements, and clinical history. As part of routine local clinical practice, prick-to-prick testing was performed using fresh raw chicken egg white equilibrated to room temperature. The procedure involved puncturing the egg white with a lancet followed by immediate application to the patient’s skin, and wheal diameters were measured in millimeters after 15 min.

#### Skin Test Reactivity Was Assessed Using Five Parameters

(1) wheal diameter obtained with commercial egg white extract (mm);
(2) wheal diameter obtained with fresh egg white prick-to-prick testing (mm);
(3) histamine wheal diameter (mm);
(4) prick/histamine ratio, calculated as the commercial egg white wheal diameter divided by the histamine wheal diameter;
(5) prick-to-prick/histamine ratio, calculated as the fresh egg white prick-to-prick wheal diameter divided by the histamine wheal diameter.

All skin testing procedures were conducted at a single center under standardized conditions by experienced personnel from the pediatric allergy and immunology team.

### 2.3. Serum Samples and In Vitro Specific IgE Measurement

Serum samples obtained from each participant were aliquoted and stored at −20 °C until analysis. Serum egg white–specific immunoglobulin E (sIgE) levels were measured using a chemiluminescent enzyme immunoassay on the IMMULITE^®^ 2000 system (Siemens Healthineers Diagnostics, Deerfield, IL, USA), in accordance with the manufacturer’s instructions. The analytical measurement range of the assay was 0.1–100 kUA/L. Component-resolved diagnostics (e.g., ovalbumin or ovomucoid) were not performed due to the retrospective study design and the unavailability of allergen component testing on the IMMULITE^®^ platform.

### 2.4. Oral Food Challenge Procedures

Oral food challenge (OFC) was performed to establish clinical reactivity to egg in accordance with European Academy of Allergy and Clinical Immunology (EAACI) food allergy guidelines. The OFC was conducted using fully cooked egg white prepared by boiling at 100 °C for at least 15 min. A standardized open challenge protocol was applied, with incrementally increasing doses administered at 15–20 min intervals according to predefined gram amounts and corresponding protein content. Dose escalation, observation intervals, stopping criteria, and safety precautions were standardized in accordance with published consensus recommendations for oral food challenges [12]. The total cumulative dose was planned to reach approximately 30 g of egg white, equivalent to about 3.5 g of egg white protein, corresponding to one whole cooked egg. Challenges were conducted under supervised hospital conditions by experienced personnel, with predefined observation intervals between each dose increment.

The oral food challenge was immediately discontinued and classified as positive (OFC-positive) upon the occurrence of objective allergic manifestations involving any organ system, including cutaneous (urticaria, angioedema), gastrointestinal (vomiting, abdominal pain, diarrhea), respiratory (cough, wheezing, dyspnea), or cardiovascular (hypotension, syncope) symptoms. In addition, among patients with atopic dermatitis, those who developed a flare of dermatitis following the reintroduction of egg into the diet during follow-up were also classified as OFC-positive.

Prior to challenge administration, all patients underwent systematic evaluation for active infection, concurrent systemic illness, and uncontrolled asthma. OFC was not performed in patients with identified contraindications. All challenges were conducted by experienced personnel in a fully equipped setting with immediate access to emergency intervention equipment, including epinephrine.

### 2.5. Data Collection

Medical records underwent systematic retrospective review, with comprehensive extraction of the following variables recorded on standardized case report forms: demographic characteristics (age, sex), presenting clinical manifestations, serum egg white-specific IgE concentrations, clinical reaction phenotypes (e.g., urticaria, atopic dermatitis), presence of concomitant atopic conditions (atopic dermatitis, asthma, allergic rhinitis), documented history of egg-associated adverse reactions, skin test parameters [SPT wheal diameter, prick-to-prick wheal diameter, and histamine wheal diameter], and oral food challenge (OFC) results (positive/negative).

Primary Endpoint: The primary outcome was OFC positivity, defined as the occurrence of objective acute allergic reactions involving any organ system during the oral food challenge.

Secondary Endpoint: Secondary outcomes included delayed clinical reactions during follow-up, particularly flares of atopic dermatitis following the reintroduction of egg into the diet.

### 2.6. Statistical Analysis

Continuous variables were assessed for normality using the Shapiro–Wilk test and are presented as median (interquartile range [IQR]) where appropriate. Between-group comparisons were performed using the Mann–Whitney U test for continuous variables and the chi-square or Fisher’s exact test for categorical variables. Correlations were assessed using Spearman’s rank correlation coefficient.

Diagnostic performance of biomarkers was evaluated using receiver operating characteristic (ROC) curve analysis, with calculation of the area under the curve (AUC), sensitivity, and specificity. Exploratory cut-off values were determined using the Youden index. Ninety-five percent confidence intervals for sensitivity and specificity were calculated using the Wilson score method.

Multivariable logistic regression analysis was performed to identify independent predictors of oral food challenge (OFC) positivity. Variables associated with OFC outcome at *p* < 0.10 in univariate analyses were considered, and absolute egg white skin prick test (SPT) wheal diameter and serum egg white–specific IgE levels were simultaneously entered into the model using the enter method based on a priori clinical relevance. Histamine-normalized indices were excluded because of collinearity with absolute wheal diameter and lack of independent predictive value. Other clinically relevant covariates (e.g., age, sex, and presenting phenotype such as atopic dermatitis/urticaria and concomitant atopic diseases) were evaluated in univariable analyses but were not retained due to lack of association and/or the limited number of OFC-positive events. Model fit was assessed using the omnibus likelihood ratio test and Nagelkerke pseudo-R^2^, and discrimination was evaluated by the AUC. Given the limited number of OFC-positive cases, internal validation (e.g., bootstrapping or cross-validation) was not performed, and the regression results should therefore be interpreted as exploratory.

A two-tailed *p*-value < 0.05 was considered statistically significant. All statistical analyses were performed using IBM SPSS Statistics for Windows (Version 25.0; IBM Corp., Armonk, NY, USA).

## 3. Results

### 3.1. Patient Characteristics and Study Population

The final study cohort comprised 105 pediatric patients, of whom 23 (21.9%) demonstrated positive oral food challenge (OFC) outcomes and 82 (78.1%) demonstrated negative outcomes. The cohort consisted of 61 males (58.1%) and 44 females (41.9%). Median age at evaluation was 17.5 months (range: 7–156 months). Baseline clinical and laboratory characteristics of patients stratified by OFC outcome are summarized in Table 1.

### 3.2. Comparison of Clinical and Laboratory Parameters Between OFC Groups

Comparative analysis revealed significant differences between OFC-positive and OFC-negative groups in several key parameters. Egg white prick wheal diameter and prick/histamine ratio were significantly elevated in the OFC-positive group (*p* < 0.001 for both). Additionally, serum egg white-specific IgE concentrations were significantly higher in patients with positive OFC results (*p* < 0.001).

In contrast, no statistically significant between-group differences were observed for age, prick-to-prick wheal diameter, or prick-to-prick/histamine ratio (*p* > 0.05 for all comparisons). Sex distribution did not differ significantly between OFC-positive and OFC-negative groups (*p* = 0.646). Urticaria was the most frequently reported presenting symptom in both groups, observed in 15 of 23 patients (65.2%) in the OFC-positive group and 43 of 82 patients (52.4%) in the OFC-negative group.

### 3.3. Correlation Analysis

Correlation analysis using Spearman’s rank correlation coefficient revealed no significant associations between age and the following skin test parameters: prick wheal diameter, prick-to-prick wheal diameter, histamine wheal response, or histamine-normalized indices (prick/histamine ratio and prick-to-prick/histamine ratio) (*p* > 0.05 for all comparisons).

In contrast, serum egg white-specific IgE concentrations demonstrated a weak-to-moderate positive correlation with age that achieved statistical significance (ρ = 0.297; *p* = 0.002). Additionally, a weak but statistically significant positive correlation was identified between prick and prick-to-prick wheal diameters (ρ = 0.277; *p* = 0.001).

### 3.4. Discriminatory Performance of Serum Egg White-Specific IgE

**Figure 1:**
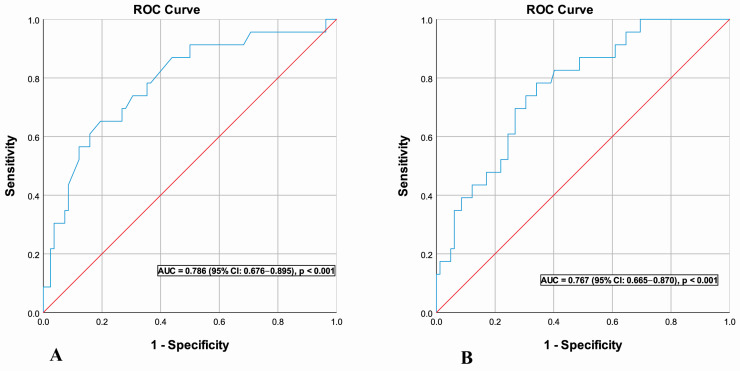
Receiver operating characteristic (ROC) curves of the egg white prick/histamine ratio (**A**) and egg white–specific IgE (**B**) for predicting oral food challenge (OFC) positivity.

ROC curve analysis for serum egg white-specific IgE concentrations yielded an area under the curve (AUC) of 0.767 (95% CI [0.665–0.870]; *p* < 0.001; Figure 1), indicating moderate-to-good discriminatory capacity for predicting OFC positivity. A ROC-derived exploratory candidate threshold of 6.35 kUA/L was identified to illustrate sensitivity–specificity trade-offs (Youden index), rather than to define a definitive diagnostic cut-off; at this level, sensitivity was 78.3% and specificity was 65.9%. Analysis of alternative threshold values revealed clinically relevant trade-offs between sensitivity and specificity. When prioritizing high sensitivity to minimize false-negative results, a candidate threshold of 1.8 kUA/L was identified, yielding a sensitivity of 95.7% with reduced specificity (35.4%). Conversely, when prioritizing high specificity (≥95%) to minimize false-positive results, a candidate threshold of 34.7 kUA/L yielded a specificity of 95.1% with substantially reduced sensitivity (21.7%). When the specific IgE/total IgE ratio was evaluated, its ability to discriminate OFC-positive and OFC-negative patients was lower than that of specific IgE alone (AUC: 0.677 vs. 0.767).

### 3.5. Discriminatory Performance of Prick/Histamine Ratio

ROC curve analysis for the prick/histamine ratio yielded an area under the curve (AUC) of 0.786 (95% CI [0.676–0.895]; *p* < 0.001), demonstrating moderate-to-good discriminatory performance for predicting clinical reactivity. This AUC value was slightly higher than that observed for serum egg white-specific IgE alone (AUC = 0.767). At a ROC-derived exploratory threshold of 0.85 (Youden index), which provided a balance between sensitivity and specificity, the prick/histamine ratio demonstrated a sensitivity of 78.3% and specificity of 64.6%. Analysis of alternative threshold values revealed clinically relevant sensitivity-specificity trade-offs. When prioritizing high sensitivity for screening purposes, a candidate threshold of 0.61 was identified, achieving a sensitivity of 95.7% at the expense of substantially reduced specificity (29.3%). Conversely, when prioritizing high specificity (≥95%) for confirmatory purposes in high-probability cases, a candidate threshold of 1.48 yielded a specificity of 96.3% with markedly reduced sensitivity (30.4%). The ROC-derived exploratory candidate thresholds for SPT parameters are summarized in Table 2.

### 3.6. Multivariable Predictive Modeling

Binary logistic regression analysis was performed to identify independent predictors of OFC positivity, with egg white SPT wheal diameter and serum egg white–specific IgE level entered as covariates (Table 3). Both variables emerged as significant independent predictors in the final model. The overall model demonstrated a statistically significant improvement over the null model containing only the constant term (Omnibus χ^2^ = 24.73; df = 2; *p* < 0.001), with a Nagelkerke pseudo-R^2^ of 0.323, indicating that approximately one-third of the variance in OFC outcomes was explained by the model. Within the multivariable framework, each 1 mm increase in egg white SPT wheal diameter was independently associated with a 1.41-fold increase in the odds of OFC positivity (adjusted OR: 1.41; 95% CI: 1.13–1.77; *p* = 0.003). Serum egg white–specific IgE remained independently associated with OFC positivity (adjusted OR: 31.86 per 100 kUA/L increase; 95% CI: 1.60–636.15; *p* = 0.023). Model discrimination and calibration were assessed using a probability threshold of 0.50. At this cut-point, the model achieved an overall classification accuracy of 84.8%, with high specificity for OFC-negative cases (96.3%) but limited sensitivity for OFC-positive cases (43.5%). This pattern suggests that while the model effectively identifies patients unlikely to react during OFC, it captures fewer than half of patients who ultimately demonstrate clinical reactivity. Although the prick-to-histamine ratio showed comparable univariable discriminatory performance, it was not retained in the multivariable model due to collinearity with absolute wheal diameter and lack of independent predictive value. The multivariable model demonstrated good discrimination, with an AUROC of 0.809 (95% CI: 0.700–0.917; *p* < 0.001).

Both egg white prick wheal diameter and prick/histamine ratio demonstrated good discriminatory performance for OFC outcomes, with comparable AUC values. However, the cut-off analysis revealed that prick wheal diameter provided slightly better specificity at balanced thresholds and more clinically usable performance across different decision strategies. Although normalization to histamine aimed to reduce interindividual variability, the prick/histamine ratio did not confer a clear diagnostic advantage over wheal diameter alone.

## 4. Discussion

Our findings demonstrate that egg white SPT wheal diameter, the prick-to-histamine ratio, and serum egg white–specific IgE levels were all associated with OFC positivity; however, in multivariable analysis, only absolute SPT wheal diameter and serum specific IgE remained independent predictors. This indicates that, despite their theoretical appeal, histamine-normalized SPT indices do not provide additional diagnostic value beyond absolute wheal measurements in routine practice. The modest explanatory capacity of the model further highlights the multifactorial nature of food allergy, emphasizing that clinical reactivity cannot be reliably predicted by a single biomarker. The young median age of the cohort and the absence of associations with basic demographic or clinical characteristics are consistent with previously reported heterogeneous findings, while the positive correlation between egg white–specific IgE and age likely reflects cumulative allergen exposure rather than disease severity [13,14].

ROC analysis demonstrated moderate-to-good discriminatory performance for serum egg white–specific IgE and the prick/histamine ratio, with different cut-off ranges prioritizing sensitivity or specificity depending on clinical intent. Lower thresholds favored screening and identification of children at low risk of reactivity, whereas higher thresholds supported a high probability of clinical allergy but with limited sensitivity. These findings support a tiered, probabilistic interpretation of ROC-derived thresholds as decision-support tools rather than absolute diagnostic criteria, in line with EAACI recommendations [1,7,11,15].

Although the prick/histamine ratio accounts for interindividual variability in skin reactivity, it did not demonstrate a clear diagnostic advantage over absolute SPT wheal diameter in terms of overall discrimination or clinically meaningful cut-off behavior. Similarly, prick-to-prick testing did not provide incremental diagnostic benefit. This may be explained by its uniform application across all patients, reducing spectrum effects, and by the biological mismatch between sensitization to raw egg proteins and OFCs performed with cooked egg, where thermal processing alters allergenicity [4,16,17,18]. Finally, the inherent variability of native allergen transfer during prick-to-prick testing may further attenuate its discriminatory capacity when uniformly applied across a heterogeneous pediatric population [19,20].

Several limitations warrant consideration. The retrospective, single-center design and limited sample size restrict generalizability and precluded age-specific analyses. Selective performance of OFCs introduces potential selection and verification bias, which may have inflated diagnostic performance estimates. In addition, the limited number of OFC-positive cases raises the possibility of model overfitting. Component-resolved diagnostics were not available, and the interval between last reaction and OFC could not be systematically retrieved, limiting interpretation of tolerance-related challenges. Despite these limitations, strengths of this study include standardized SPT application, OFCs conducted according to EAACI recommendations, and direct head-to-head comparison of absolute and histamine-normalized SPT parameters. 

## 5. Conclusions

Overall, our findings demonstrate that normalization of SPT responses does not confer additional diagnostic benefit over absolute wheal diameter and should be interpreted as a complementary, rather than substitutive, tool within individualized clinical decision-making. Future prospective, multicenter studies incorporating component-resolved diagnostics and age-stratified analyses are needed to refine diagnostic algorithms and optimize selective OFC use.

## Figures and Tables

**Table 1:** Baseline Clinical and Laboratory Characteristics of Patients Stratified by OFC Outcome.

Variable	OFC-Negative (*n* = 82)	OFC-Positive (*n* = 23)	*p*-Value
Demographics			
Age (months), median (IQR25–IQR75)	18.5 (13.0–33.5)	15.0 (13.0–24.0)	0.236
Sex, n (%)			0.433
Male	46 (56.1)	15 (65.2)	
Female	36 (43.9)	8 (34.8)	
Clinical Presentation			
Presenting symptom, n (%)			0.324
Urticaria	43 (52.4)	15 (65.2)	
Atopic dermatitis	35 (42.7)	6 (26.1)	
Other *	4 (4.9)	2 (8.7)	
Laboratory Parameters			
Serum specific IgE (kUA/L), median (IQR25–IQR75)	3.51 (1.07–10.73)	14.0 (7.10–33.13)	<0.001
Serum Total IgE (kU/L), median (IQR25–IQR75)	55.5 (25.6–173.5)	72.0 (51.1–395.0)	0.081
Serum specific IgE/ Total IgE, median (IQR25–IQR75)	0.07 (0.03–0.15)	0.16 (0.05–0.46)	0.010
Skin Test Parameters			
Prick wheal diameter (mm), median (IQR25–IQR75)	4.5 (3.5–6.0)	7.0 (5.0–9.0)	<0.001
Prick-to-prick wheal diameter (mm), median (IQR25–IQR75)	10.0 (6.0–12.5)	11.0 (7.0–15.5)	0.206
Histamine wheal diameter (mm), median (IQR25–IQR75)	6.0 (5.0–8.0)	5.5 (5.0–7.5)	0.570
Prick/histamine ratio, median (IQR25–IQR75)	0.74 (0.55–1.00)	1.20 (0.86–1.50)	<0.001
Prick-to-prick/histamine ratio, median (IQR25–IQR75)	1.41 (1.00–1.92)	1.70 (1.00–2.58)	0.188

Data are presented as median (IQR) or n (%). OFC, oral food challenge; IQR, interquartile range. * Includes diarrhea and vomiting.

**Table 2:** ROC-derived exploratory candidate thresholds illustrating potential sensitivity–specificity trade-offs for egg white SPT parameters.

Parameter	AUC (95% CI)	Cut-Off Strategy	Cut-Off Value	Sensitivity % (95% CI)	Specificity % (95% CI)
Prick wheal diameter (mm)	0.775 (0.662–0.887)	Balanced (Youden)	≥5.5	73.9 (52.8–88.9)	74.4 (63.8–82.9)
		High sensitivity	≥3.25	100.0 (85.7–100.0)	19.5 (12.1–29.9)
		High specificity	≥8.75	34.8 (19.3–54.6)	95.1 (87.8–98.4)
Prick/histamine ratio	0.786 (0.676–0.895)	Balanced (Youden)	≥0.90	73.9 (52.8–88.9)	69.5 (58.8–78.4)
		High sensitivity	≥0.35	95.7 (79.0–99.2)	4.9 (1.9–11.8)
		High specificity	≥1.43	30.4 (16.0–49.1)	95.1 (87.8–98.4)

**Table 3:** Multivariable Logistic Regression Analysis for Predictors of OFC Positivity.

Variable	Adjusted OR	95% CI	*p*-Value
Prick wheal diameter (per 1 mm increase)	1.41	1.13–1.77	0.003
Serum egg white–specific IgE (per 100 kUA/L increase)	31.86	1.60–636.15	0.023

OR, odds ratio; CI, confidence interval; OFC, oral food challenge. Serum egg white–specific IgE values were scaled by dividing by 100 prior to regression analysis to improve interpretability of odds ratios. Model χ^2^ = 24.73, *p* < 0.001; Nagelkerke R^2^ = 0.323. Model AUROC (95% CI): 0.809 (0.700–0.917).

## Data Availability

Data are available from the corresponding author upon reasonable request.
